# Creatine Revealed Anticonvulsant Properties on Chemically and Electrically Induced Seizures in Mice

**Published:** 2016

**Authors:** Hamed Shafaroodi, Farnaz Shahbek, Mehrdad Faizi, Farzad Ebrahimi, Leila Moezi

**Affiliations:** a *Department of Pharmacology and Toxicology, Pharmaceutical Sciences Branch, Islamic Azad University, Tehran, Iran. *; b *Department of Pharmacology, School of Medicine, Tehran University of Medical Sciences, Tehran, Iran. *; c *Department of Pharmacology and Toxicology, Faculty of Pharmacy , Shahid Beheshti University of Medical Sciences , Tehran , Iran. *; d *Department of Anesthesiology, Advocate Illinois Masonic Medical Center, University of Illinois, Chicago, IL, USA. *; e *Department of Pharmacology, School of Medicine, Shiraz University of Medical Sciences, Shiraz, Iran.*

**Keywords:** Creatine, Seizure, Pentylenetetrazole, Electroshock, Mice

## Abstract

Creatine exerts beneficial effects on a variety of pathologies in which energy metabolism and oxidative stress play an etiological role. Creatine supplements have shown beneficial effects on neurological disorders including Parkinson׳s disease, Huntington›s disease, amyotrophic lateral sclerosis, as well as Alzheimer›s disease and stroke. However, the potential benefits of creatine for patients with convulsive disorders remain poorly defined. While some authors did not suggest any anti- or pro-convulsant roles for creatine treatment, others suggest that creatine may be an anticonvulsant agent. In this study, we investigated the effects of creatine on seizures in mice.

Three models were used to explore the role of creatine on seizures in mice including intravenous pentylenetetrazole (PTZ), intraperitoneal PTZ, and electroshock models.

Acute creatine treatment (10, 20, 40 and 80 mg/Kg) significantly increased the clonic seizure threshold in the intravenous PTZ model. Sub-chronic administration of creatine (10 and 20 mg/Kg) revealed a significant anticonvulsant effect in intravenous PTZ model. Acute creatine administration (10, 20 and 40 mg/Kg) significantly decreased the frequency of clonic seizures in the intraperitoneal PTZ model. Besides, acute creatine (40 and 80 mg/Kg) decreased the incidence of tonic seizures after electroshock.

In conclusion, creatine exerts anticonvulsant effects in three seizure models; therefore, it may act as a potential drug to help patients with convulsions. However, further investigations should be done to clarify these results more.

## Introduction

According to a paper using a meta-analytic approach, more than 65 million people worldwide suffer from epileptic disorders (1). The rate of epilepsy in developed countries is approximately 50 per 100,000 individuals per year with the highest rates in the neonatal periods and in the elderly (2). While single-drug therapy provides optimal seizure control in about 80% of all patients, seizure activity remains uncontrolled in a significant number of individuals, even with the use of combination therapy (3). Therefore, further investigations to find alternative therapies for epilepsy are necessary.

Creatine (N-[aminoiminomethyl]- N-methyl glycine, Cr) is a guanidine compound synthesized from glycine, arginine, and S-adenosylmethionine in the kidney, liver, pancreas, and brain, but it can also be obtained through diet, especially from meat and fish (4, 5). Its administration increases intracellular stores of both Cr and its phosphorylated form, phosphorylcreatine (PCr), in several tissues including skeletal muscles and the brain (6, 7). Cr is a popular dietary supplement that is used by athletes in order to increase anabolic hormonal response, muscle mass, strength, and sport performance (8). 

 However, new uses for Cr have emerged suggesting that it may be important in preventing or delaying the onset of neurodegenerative diseases associated with aging. Cr has been shown to have antioxidant properties, reduce mental fatigue, protect the brain from neurotoxicity, and improve neurological disorders like depression and bipolar disorder. Therefore, the use of oral Cr supplementation is now being extended in medical fields in order to prevent or treat a number of age-related diseases, such as Parkinson›s disease,Huntington›s disease, amyotrophic lateral sclerosis, long-term memory impairments associated with progression of Alzheimer׳s disease and stroke (9-11).

Although there is increasing evidence supporting the use of Cr in treatment of several neurological diseases, the potential benefits of this compound for patients with convulsive disorders have not been clearly defined. While some authors did not suggest any anti- or pro-convulsant roles for Cr treatment (8i12), others suggest that Cr may be an anticonvulsant agent (3, 13, 14). Therefore, in the current study we studied the effects of Cr in modulating seizure propensity using different experimental models including intravenous pentylenetetrazole (PTZ), intraperitoneal PTZ,and maximal electroshock models.

## Experimental

*Chemicals*

PTZ and Cr were purchased from Sigma-Aldrich (USA) and both drugs were dissolved in physiological saline solution to a concentration at which the required doses were administered in a volume of 10 mL/Kg. Cr was administered intraperitoneally. PTZ was injected intraperitoneally or intravenously depending on model of induced seizure. 

*Animals*

Male NMRI albino mice weighing 20-30 g were housed 5-6 per cage in a room with a controlled temperature of 22 ± 1 °C. The room had an alternating 12 h light-dark cycle starting at 7:00 A.M. Animals had free access to food and water and were group housed during testing procedures. All procedures were approved and carried out in accordance with institutional guidelines for laboratory animal care and use. Each mouse was used only once and each treatment group consisted of 8-10 animals, which were randomly selected. All animals were acclimated at least 3 days before experiments. All behavioral experiments were conducted during the period between 10:00 and 14:00 hours. All the animals were euthanized immediately after the experiment.

*Behavioral seizure evaluation*

*Intraperitoneal PTZ-induced seizure*

Generalized tonic-clonic seizure induced by intraperitoneal pentylenetetrazole is a distinct model related to generalized tonic-clonic seizures (15). In this model, pentylenetetrazole (85 mg/Kg, CD97 for generalized tonic-clonic seizures in the current experiment) was administered with a single intraperitoneal injection (16, 17). Immediately after injection of PTZ, animals were transferred to an open field (50 cm in diameter) and monitored for the appearance of convulsion or death in 30 min. Following the administration of 85 mg/Kg of PTZ, time latencies and incidences for the generalized clonus were reported. Generalized clonus is described as the involvement of all four limbs and tail rearing, wild running and jumping, sudden loss of upright posture and autonomic signs such as hyper-salivation and defecation. The incidence of death was also recorded. 

*Intravenous PTZ-induced seizure*

In addition to intraperitoneal PTZ-induced seizure model, drug effects on clonic-tonic seizures can be studied by using intravenous PTZ-induced seizure model which induces tonic seizures in all animals. Compared to the intraperitoneal injection of a fixed dose of PTZ in a group of animals, PTZ can be used by intravenous infusion to determine the individual seizure threshold of each subject. This model may have advantages, because thresholds for clonic and tonic seizures can be separately determined in the same animals, thus providing a sensitive test system for separate evaluation of drug effects on different seizure types in a small number of rodents (15, 18). In this study, PTZ-induced clonic seizure threshold was determined by inserting a 30 gauge dental needle into the tail vein of the mouse and securing the needle with a narrow piece of adhesive tape (16). PTZ solution (0.5%) was infused at a constant rate of 0.5 mL/min using an infusion pump (Harvard, USA) to unrestraine freely moving animals. Infusion was halted when forelimb clonus followed by full clonus of the body was observed. Minimal dose of PTZ (mg/Kg of mice weight) needed to induce clonic seizure was considered as an index of seizure threshold.

*Maximal electroshock test*

To examine the anticonvulsant effects of Cr, an electroconvulsive therapy apparatus (Model 7800, UgoBasile, Camerio, Italy) was also used. In maximal electroshock (MES) test, passing alternating current (50 Hz, 35 mA and 0.2 s) via ear electrodes causes tonic extensions of the hind limbs of mice. In order to improve electrode contact, the electrodes were moistened with normal saline before being attached to the ears of mice (19, 20). Data were expressed as percent protection, which is the percentage of animals in each group that did not exhibit hind-limb extension after electroshock.

*Treatment*

Three methods of intraperitoneal and intravenous administration of PTZ and electroshock were used to assess the seizure susceptibility.

Intravenous PTZ experiments: In experiment 1, to find out the best time for anticonvulsant effect of the drug, Cr (20 mg/Kg) was administered intraperitoneally, 30, 60 and 120 min prior to PTZ to distinct groups of mice. In experiment 2, Cr (5, 10, 20, 40 and 80 mg/Kg) was administered intraperitoneally in different groups of male mice 60 min before intravenous PTZ. This time point was selected according to experiment 1. In experiment 3, which we wanted to find the sub-chronic effects of Cr, the drug was administered at doses of 10 and 20 mg /Kg intraperitoneally for 5 days and intravenous PTZ experiments were done in the sixth day.

Intraperitoneal PTZ experiments: Cr (10, 20 and 40 mg/Kg) was administered intraperitoneally in different groups of male mice 60 min before intraperitoneal PTZ.

Electroshock experiments: Cr (20, 40 and 80 mg/Kg) was injected intraperitoneally in different groups of mice 60 min before electroshock.

*Statistical analysis*

Data are expressed as means ± SEM of 8-10 mice and analyzed using the SPSS statistical software package (Version 18.0). One-way analyses of variance (ANOVA) and post hoc Tukey’s tests were used to analyze the data. In order to determine the protective effects against tonic seizure and death, Fisher’s exact test was used. *P*<0.05 was considered statistically significant.

## Results

*Effect of acute and sub-chronic creatine administration on clonic seizure threshold in intravenous PTZ model*

**Figure 1:**
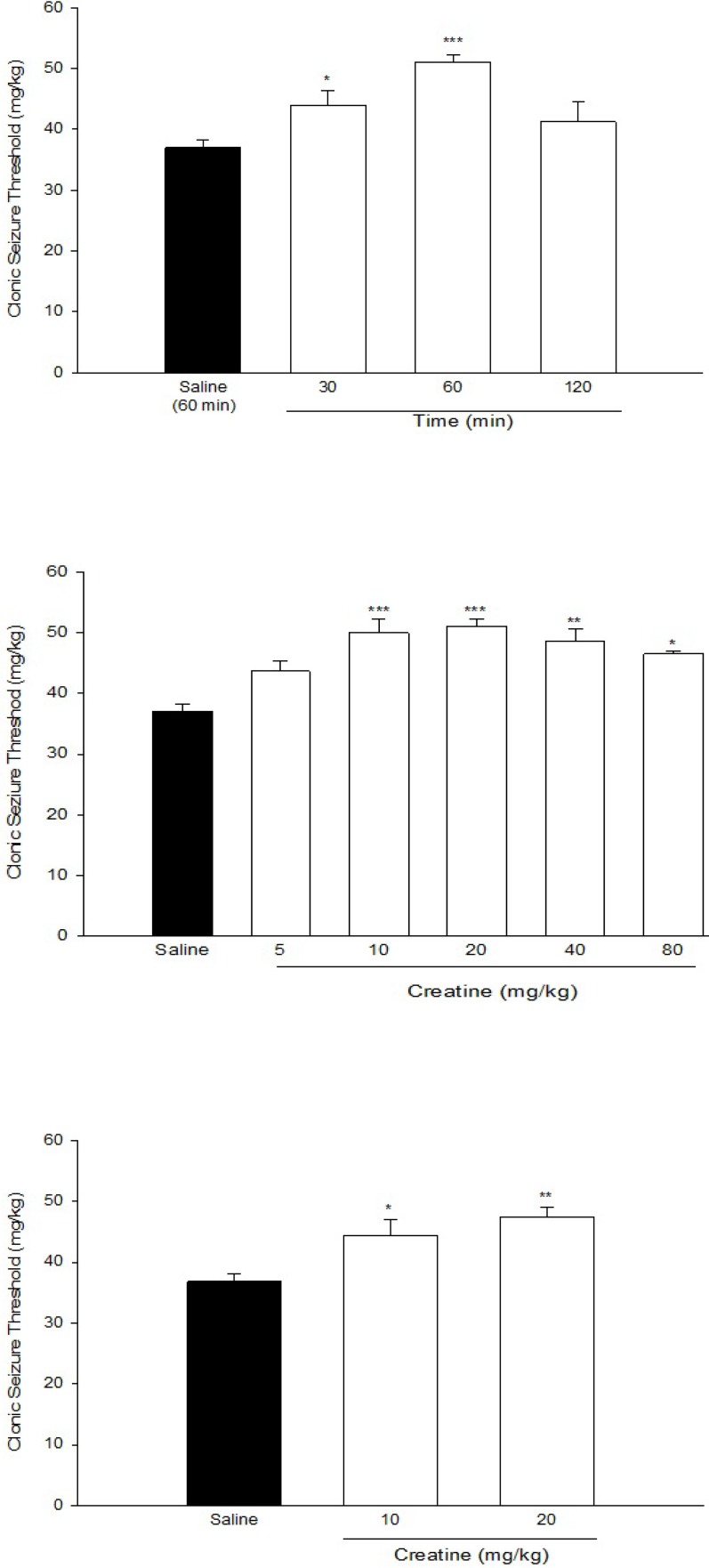
(a) Time course of the anticonvulsant effect of creatine (20 mg/Kg) (b) Effect of acute creatine treatment (c) Effect of sub- chronic creatine treatment on intravenous PTZ-induced seizure threshold in mice. Creatine was administered intraperitoneally. Data are means ± SEM. *P < 0.05, **P < 0.01 and ***P < 0.001 compared with the saline group. Each group consisted of eight to ten mice

Figure 1a. shows anticonvulsant effect of Cr (20 mg/Kg) at different times after injections. One-way ANOVA revealed a significant effect of Cr in different times (F[3,23] = 19.577, *P* < 0.001). Post hoc analysis indicated that Cr exerted an anticonvulsant effect 30 min after administration (*P *< 0.05). This effect increased thereafter at 60 min after Cr administration, which was its maximal effect (*P* < 0.01).Therefore, we administered Cr, 60 min before seizure induction in the next experiments.

Figure 1b. shows the effect of acute intraperitoneal administration of different doses of Cr (5, 10, 20, 40, and 80 mg/Kg) on PTZ-induced clonic seizure threshold. One-way ANOVA revealed a significant effect of treatment (F[5,37] = 9.897, *P* < 0.001). Post hoc analysis indicated a significant anticonvulsant effect of acute Cr treatment at doses of 10, 20, 40, and 80 mg/Kg with maximal effect at 10 and 20 mg/Kg (*P* < 0.001).

**Figure 2:**
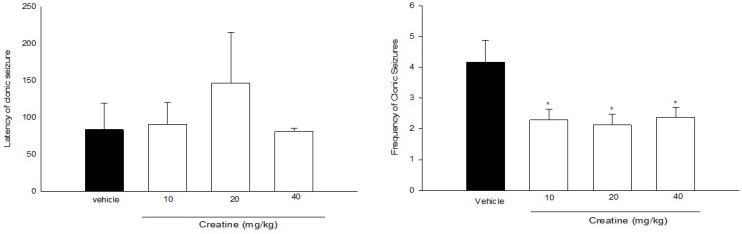
Effect of acute treatment with different doses of creatine on (a) clonic seizure threshold and (b) frequency of clonic seizures in intraperitoneal PTZ mode of mice. Creatine was administered intraperitoneally 60 min prior to PTZ injection. Data are expressed as mean ± S.E.M. *P < 0.05 compared to saline group. Each group consisted of eight to ten mice

**Table 1:** Effect of acute treatment with different doses of creatine on the incidence of death in intraperitoneal PTZ-induced seizure model

**Groups**	**Mortality (%)**
Vehicle (saline)	83.3
Creatine (10 mg/Kg)	75
Creatine (20 mg/Kg)	50
Creatine (40 mg/Kg)	37.5

Percentage of death subsequent pentylenetetrazole was compared with saline group using Fisher-exact test (P > 0.05). Each group of mice consisted of eight to ten animals.

**Table 2:** Effect of acute treatment with different doses of creatine on the incidence of hind-limb extension in electroshock-induced seizure model

**Groups**	**Mortality (%)**
Vehicle (saline)	83.3
Creatine (10 mg/Kg)	75
Creatine (20 mg/Kg)	50
Creatine (40 mg/Kg)	37.5

Percentage of hind-limb extension subsequent electroshock was compared with saline group using Fisher-exact test (*P < 0.05). Each group of mice consisted of eight to ten animals.

Figure 1c. shows the effect of sub-chronic administration of Cr (10 and 20 mg/Kg) on intravenous PTZ-induced clonic seizure threshold. One-way ANOVA revealed a significant anticonvulsant effect of sub-chronic Cr administration at both doses [F(2, 19) = 9.22, *P* < 0.01].

*Effect of acute administration of different doses of creatine on intraperitoneal PTZ-induced seizures*

Figure 2a. illustrates the effect of acute administration of Cr (10, 20, and 40 mg/Kg) 60 min prior to intraperitoneal PTZ administration on clonic seizure latency. One-way ANOVA did not reveal a significant effect of Cr on clonic seizure latencies [F(3, 26) = 0.556, *P* > 0.05]. 

The frequency (number of clonic seizures within 30 min after PTZ administration) has been shown in Figure 2b. [F(3, 26) = 3.064, *P* < 0.05]. Cr (10, 20 and 40 mg/Kg) significantly decreased the frequency of clonic seizures (*P* < 0.05). However, it did not have any significant effect on mortality rate in all groups (Table 1).

*Effect of acute administration of different doses of creatine on electroshock-induced seizures*

The results of acute administration of different doses of Cr (20, 40 and 80 mg/Kg) in the electroshock-induced seizure model are shown in Table 2. Fisher’s exact test revealed a significant effect of Cr (40 and 80 mg/Kg) on the incidence of tonic seizures after electroshock (*P* < 0.05).

## Discussion

The present work shows the anti-convulsant effect of Cr in three seizure models including intravenous PTZ, intraperitoneal PTZ, and electroshock. In addition to the pool of Cr in the muscle, high levels of Cr are found in the brain (21). Although many of the molecular mechanisms are not well understood, Cr supplementation has been proposed and/or proven partially in a variety of animal/cellular models of neurodegenerative diseases such as Alzheimer’s, Parkinson’s, and Huntington’s disease (22). There are some papers which showed the anti-convulsant effects of creatine. For example, it has been shown that the acute creatine administration improves mitochondrial membrane potential and protects rats against pentylenetetrazole-induced seizures (14). Another study (Magni *et al*., 2007) reported that creatine decreases convulsions and neurochemical alterations induced by glutaric acid in rats (13). On the other hand, some authors did not show any effect of Cr on convulsion. For example, Mikati *et al*. (2004) showed that creatine supplementation cannot prevent spontaneous recurrent seizures (12) and Saraiva *et al*. (2012) indicated that creatine did not protect against seizure susceptibility after severe traumatic brain injury (23).

Induction of seizures by intravenous infusion of PTZ is a standard experimental model of clinical myoclonic seizures that has both face and construct validity (15, 24). This model has proven to be more sensitive than intraperitoneal PTZ administration and allows better detection of modulatory effects on convulsive tendency (15, 25, 26). PTZ increases activity in major epileptogenic centers of the forebrain like amygdala and piriform cortex (27). Neurochemical evidence suggests that PTZ binds to the picrotoxin site of the GABA receptor complex and blocks GABA-mediated inhibition (28). In this study, we indicated a significant anticonvulsant effect of acute Cr treatment at doses of 10, 20, 40 and 80 mg/Kg with maximal effect at 10 and 20 mg/Kg in intravenous PTZ model. We also showed a significant anticonvulsant effect of sub-chronic Cr administration (10 and 20 mg/Kg, for seven days) in intravenous PTZ models. To the best of our knowledge, this study is the first study that showed the anti-convulsant effect of Cr in intravenous PTZ model. We also examined the effect of acute Cr treatment on generalized tonic–clonic seizures induced by intraperitoneal injection of high dose of PTZ. Generalized tonic-clonic seizure induced by intraperitoneal high dose of PTZ is an animal model related to grand mal seizures in humans (15, 29). In this model, PTZ (85 mg/Kg, CD97 for clonic seizures in the current experiment) was administered with a single intraperitoneal injection to evaluate the latency for the onset of clonic seizures and the incidence of tonic and death following seizures (16, 17). In intraperitoneal PTZ model we revealed that Cr at doses of 10, 20, and 40 mg/Kg/day significantly decreased the frequency of clonic seizures without any effect on clonic seizure latency and death. The anti-convulsant effects of Cr in acute treatment which shows in PTZ seizure models are in line with some previous studies (13, 14). Rambo *et al*., 2013 showed that acute Cr treatment prevents the increase in electroencephalographic wave amplitude typically elicited by intraperitoneal PTZ. Cr treatment also increases the latency periods of first myoclonic jerks, lengthened the latency periods of the generalized tonic–clonic seizures and reduces the time spent in the generalized tonic–clonic seizures induced by PTZ (14). 

Apart from PTZ seizure models, in this study another seizure model, the maximal (tonic extension) electroshock seizure (MES) test, a model for generalized tonic-clonic (grand mal) seizures was used which is almost universally employed today in anticonvulsant screening, structure-activity research, ‘rational drug design’, and other approaches to antiepileptic drug development (30). We believe that this is the first study revealed a significant effect of Cr (40 and 80 mg/Kg) on the incidence of tonic seizures after electroshock. Based on our findings, Cr might play a role (as a drug or as a food additive) in people with seizure. 

In conclusion, we showed the anti-convulsant effects of Cr in 3 animal models of seizure including intravenous PTZ, intraperitoneal PTZ, and maximal electroshock.
